# RPpocket: An RNA–Protein Intuitive Database with RNA Pocket Topology Resources

**DOI:** 10.3390/ijms23136903

**Published:** 2022-06-21

**Authors:** Rui Yang, Haoquan Liu, Liu Yang, Ting Zhou, Xinyao Li, Yunjie Zhao

**Affiliations:** Department of Physics, Institute of Biophysics, Central China Normal University, Wuhan 430079, China; ryang@mails.ccnu.edu.cn (R.Y.); liuhaoquan@mails.ccnu.edu.cn (H.L.); YL2019212120@mails.ccnu.edu.cn (L.Y.); tzhou@mails.ccnu.edu.cn (T.Z.); lixinyao@mails.ccnu.edu.cn (X.L.)

**Keywords:** RNA–protein interaction, pocket database, interaction mechanism

## Abstract

RNA–protein complexes regulate a variety of biological functions. Thus, it is essential to explore and visualize RNA–protein structural interaction features, especially pocket interactions. In this work, we develop an easy-to-use bioinformatics resource: RPpocket. This database provides RNA–protein complex interactions based on sequence, secondary structure, and pocket topology analysis. We extracted 793 pockets from 74 non-redundant RNA–protein structures. Then, we calculated the binding- and non-binding pocket topological properties and analyzed the binding mechanism of the RNA–protein complex. The results showed that the binding pockets were more extended than the non-binding pockets. We also found that long-range forces were the main interaction for RNA–protein recognition, while short-range forces strengthened and optimized the binding. RPpocket could facilitate RNA–protein engineering for biological or medical applications.

## 1. Introduction

RNA–protein complexes play irreplaceable roles in biological processes, including gene regulation, protein synthesis, and virus replication [1,2,3]. For example, the ribosome is a large RNA–protein complex for protein synthesis [4]. RBPs bind the target RNA to form ribonucleoprotein complexes and perform biological functions [5,6,7]. For example, the SARS-CoV-2 S protein stabilizes the virus RNA and enhances virus translation by hijacking the host factor IGF2BP1 [8]. Thus, the RNA–protein structural pocket information helps understand biological processes and drug design [9,10,11].

Currently, the available RNA–protein databases can be divided into three categories. (1) The structural RNA–protein database. The Nucleic Acid Database (NDB) and Protein Data Bank (PDB) provide experimentally determined RNA–protein complexes with sequences and structures [12,13]. In addition, the 3D shapes of the complex assemblies help researchers to understand structure and function principles. (2) The comprehensive RNA–protein database. The RISE, RNAInter, DBBP, and NPInter integrate multiple resources and provide the sequences, secondary structures, and annotations on the interaction interface [14,15,16,17]. (3) The RNA–protein docking database. The DM-RPIs, RPI-Pred, NPDOCK, HDOCK, and RNAct provide RNA–protein structures with weakly homologous complexes [18,19,20,21,22]. However, none of the available RNA–protein databases provide detailed analyzed interaction features. Especially, pocket-based information is needed for RNA–protein interaction mechanism understanding at a molecular level.

We propose a pocket-based interaction database, RPpocket, for RNA–protein complexes. We first identify the RNA–protein interaction interface and binding sites. Then, we systematically analyze the secondary structures and topological features for the interface interaction. The results show that the protein loop prefers to bind in the RNA stem region. The electrostatic interactions are the main force for long-range recognition, while short-range forces optimize and strengthen the interactions. RPpocket provides a straightforward framework to reveal RNA–protein recognition principles for RNA-related drug development or medical applications. 

## 2. Results

### 2.1. Overview of the RPpocket Database

**Figure 1:**
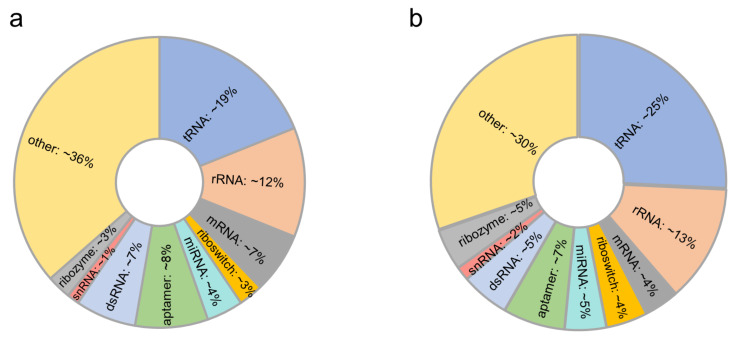
(**a**,**b**) the proportion of various types of RNA and the pocket distribution of each RNA type in our dataset.

There are 74 non-redundant RNA–protein structures and 793 pockets in the RPpocket database. The RNA length is 20~266 nucleotides, and the protein length is 22~477 amino acids. According to the different functions, we divided these 74 structures into the following categories (Figure 1 and Table S1): tRNA (14), rRNA (9), mRNA (5), riboswitch (2), microRNA (3), aptamer (6), dsRNA (5), snRNA (1), ribozyme (2), and other (27). Unlike most databases that only contain sequence and structure information, RPpocket provides more detailed interaction information with binding and topological characteristics.

### 2.2. Characteristics of Binding Motifs

**Figure 2:**
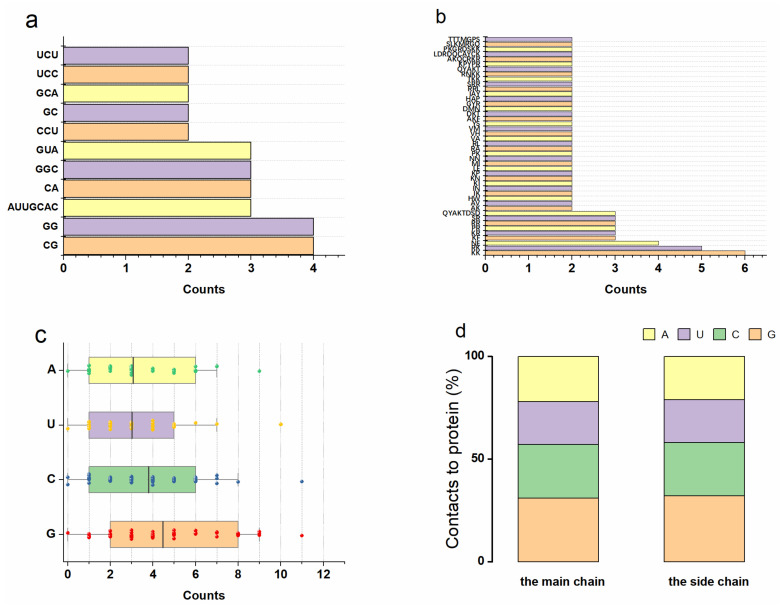
(**a**,**b**) The statistical results of the RNA/protein-binding fragments. (**c**) The RNA nucleotide distributions located at the RNA–protein interface. The distributions of G (31.3%) and C (26.4%) are significantly higher than A (21.5%) and U (20.8%). (**d**) The number of the RNA nucleotide backbone (phosphate and sugar) and side-chain (base) interactions with proteins.

The sequence features of binding sites can help us understand the RNA–protein interaction mechanism. We focused on the RNA–protein interactions formed by single RNAs and single proteins to avoid the induced structural changes of multi-body interactions. Here, we retained the binding fragments with length ≥ 2 nt/aa, which led to 149 RNA- and 285 protein-binding sites. In addition, we screened the binding fragments, which appeared at least twice. Thus, 11 RNA- and 44 protein-binding fragments are frequently involved in RNA–protein interactions (Figure 2a,b). It is noted that ‘CG’ and ‘GG’ were the most favorable binding fragments in RNA while ‘KR’ was the most favorable binding fragment in protein. We further investigated the nucleotide distributions (Figure 2c,d). The distributions of G (31.14%) were significantly higher than C (26.41%), A (21.42%), and U (21.03%). G formed interactions with amino acids more easily due to its double-ring side-chain chemical structure.

**Figure 3:**
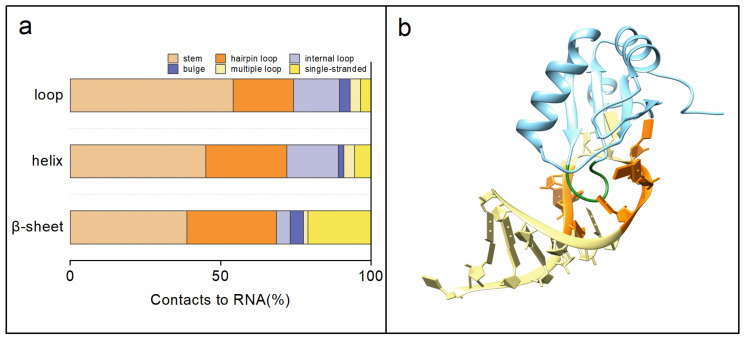
(**a**) The secondary structure distributions at the RNA–protein interaction interface. The protein loops prefer to bind to the RNA stem region. (**b**) An example of a protein loop binding to an RNA stem region (PDB code: 1URN). Green and orange represent the binding interface between protein and RNA.

In the previous research, Wang et al. analyzed two RNA–ligand datasets for binding mechanisms. One RNA–ligand dataset was constructed by Wang et al. [23], and the other RNA–ligand dataset was built by Philips et al. [24]. The results showed that 98% of nucleotides were located at or near the loop regions, suggesting that secondary structures can identify binding features [23]. Therefore, we identified and analyzed 137 RNA secondary structure fragments and 312 protein secondary structure fragments involved in RNA–protein interactions. The sequence and secondary structure information for RNAs are presented in Table S2. Table S3 shows the statistical secondary structure analysis for RNA–protein interactions. The stem region is the favorite secondary structure motif for RNA–protein interactions in rRNA, tRNA, aptamer, and others. However, the hairpin loop and internal loop are the favorite secondary structure motifs for RNA–protein interactions in riboswitch and mRNA. The loop region is the favorite secondary structure motif for RNA–protein interactions for proteins. Overall, the stem region is the most favorite motif in RNA secondary structures, followed by the hairpin loop, internal loop, single-stranded, bulge, and multiple loops. At the protein interaction interface, the secondary structure motifs are most commonly in the loop region, followed by the helix region, and the β-sheet region (Figure 3a). The structural views show that the protein loops prefer to interact with the major groove in the RNA stem (Figure 3b).

**Figure 4:**
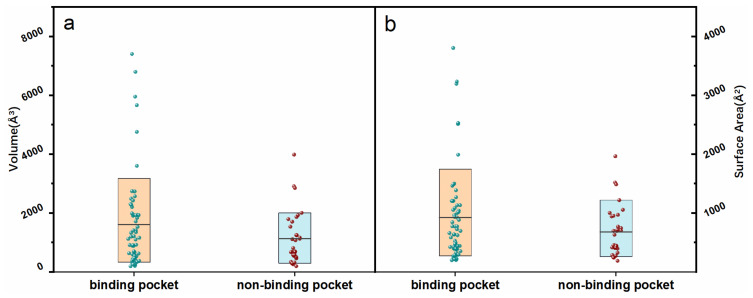
The geometric information distribution of surface area (**a**) and volume (**b**) for RNA-binding pockets and -non-binding pockets. Boxes in (**a**,**b**) represent the 80% core distributions (ranging from 10th to 90th percentile). The black lines on the box represent the average value.

The pocket geometrical volume and surface area information are some of the most critical characteristics of RNA–protein interactions. We analyzed the topological features of the RNA pockets, including 60 binding and 32 non-binding pockets. The volume, surface area, effective radius, sphericity, and pocket centroid were calculated and recorded in our database. We performed a statistical pocket geometrical analysis for different RNA categories (Table S4). The results showed that the binding pockets’ volume and surface area were larger than the non-binding pockets in rRNA, riboswitch, tRNA, and microRNA. However, the volume and surface area of the binding pockets were smaller than the non-binding pockets in aptamer. Figure 4 indicates that the binding pockets (volume of 1670.03 Å^3^; surface area of 923.77 Å^2^) were more extended than the non-binding pockets (volume of 1122.91 Å^3^; surface area of 678.94 Å^2^).

### 2.3. The RNA–Protein Interaction Mechanism

**Figure 5:**
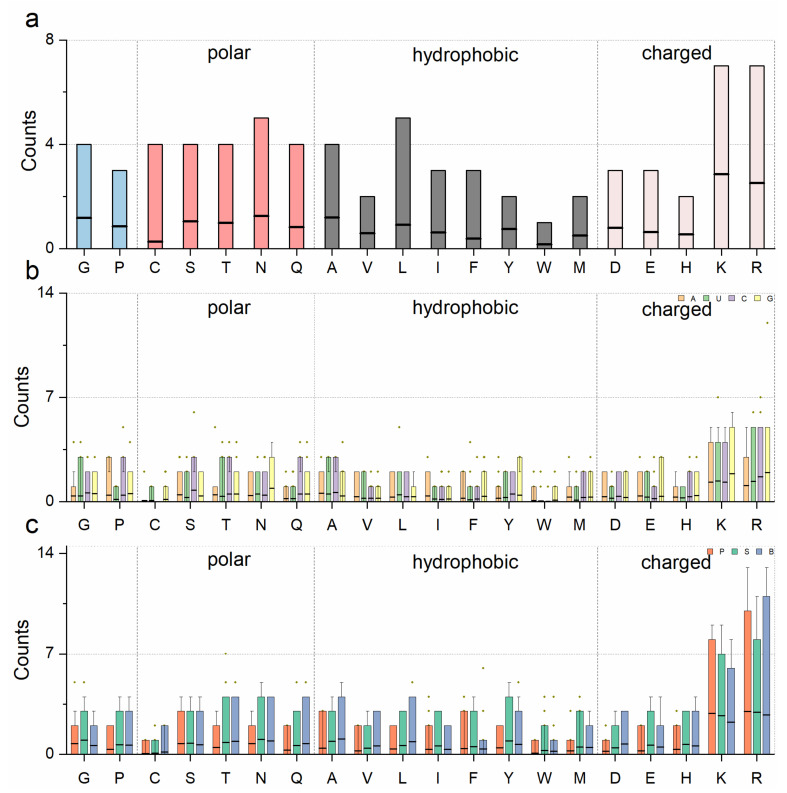
(**a**) The average frequency of the amino acids located at the RNA–protein interface. (**b**) The average contribution of the amino acids interacting with nucleotides. (**c**) The average contribution of the amino acids interacting with RNA backbone (phosphate and sugar) and bases. Boxes in (**a**–**c**) represent the 90% core distribution (ranging from the 5th to 95th percentile). The black lines on the box represent the average values.

**Figure 6:**
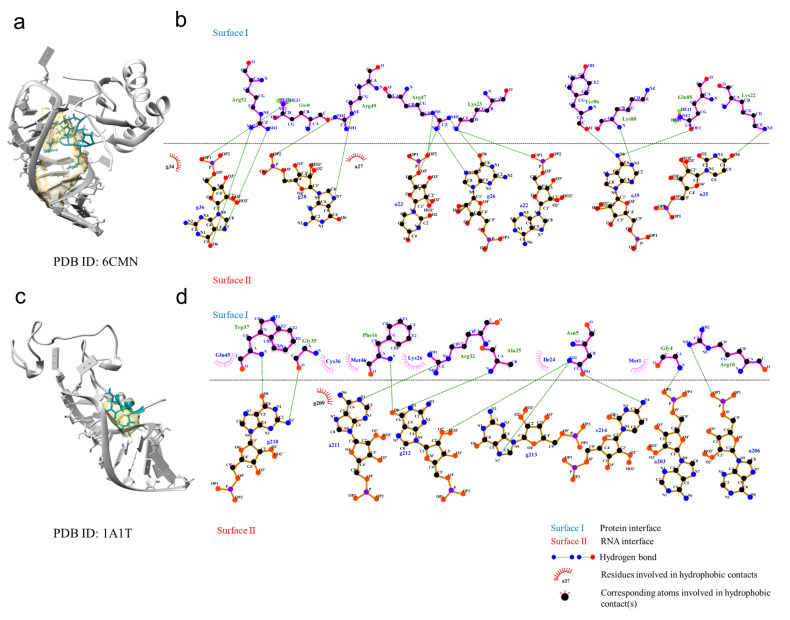
Examples of RNA-binding pocket proteins. (**a**) The structure of HIV-1 TAR RNA binds to the RNA recognition motif (PDB ID: 6CMN). (**b**) Hydrogen bond and hydrophobic contacts for the RNA–protein interface (PDB ID: 6CMN). (**c**) The structure of HIV-1 nucleocapsid protein binds to the SL3 psi-RNA (PDB ID: 1A1T). (**d**) Hydrogen bond and hydrophobic contacts for the RNA–protein interface (PDB ID: 1A1T).

To understand RNA–protein interaction patterns, we calculated the binding frequency between the nucleotides and amino acids of the representative structures in the RPpocket database. There were more positive amino acids (histidine, lysine, and arginine) involved in the interactions (31.48%) than negative amino acids (aspartic and glutamic, 7.57%). Moreover, the proportions of hydrophobic amino acids (alanine, valine, leucine, isoleucine, phenylalanine, tyrosine, tryptophan, and methionine), polar amino acids (cysteine, serine, threonine, asparagine, and glutamine), and other amino acids (glycine and proline) were 26.94%, 23.21%, and 10.80%, respectively (Figure 5a). We further divided the nucleotides into the backbone (phosphate and sugar) and side chain to explore the interaction characteristics. Figure 5b,c show that the positive amino acids have the highest probability in all interactions. The phosphate group also showed a higher probability than the sugar and side chain. Finally, we analyzed the hydrogen bonds and hydrophobic interactions using Ligpolt+ [25,26]. Figure 6a,b show that HIV-1 TAR RNA binds to the RNA recognition motif (RRM) (PDB ID: 6CMN). Fourteen hydrogen bonds and two hydrophobic contacts were involved in this RNA–protein interaction. Moreover, there were also 14 electrostatic interactions between HIV-1 TAR RNA binding with RRM. It is noted that the β2-β3 loop of the RRM was inserted into the pocket of the RNA stem region. In another example (Figure 6c,d, PDB ID:1A1T), the HIV-1 nucleocapsid protein bound to the SL3 psi-RNA and showed similar characteristics. The results indicate that long-range electrostatic interactions bring RNA and protein together. Then, the short-range interactions optimize the complex.

### 2.4. The Advantages of the RPpocket Database

In general, RPpocket has the following characteristics and advantages: (1) containing 132 RNA pockets and 661 protein pockets extracted from non-redundant 74 RNA–protein complexes; (2) providing the sequence and tertiary structure of all complexes in the dataset; (3) showing the interaction patterns of binding sites and secondary structures between RNA and protein; (4) analyzing the topological pocket features (surface area, volume, effective radius, spherical similarity score, etc.); (5) allowing the users to rotate and translate the structure in the visualization module; (6) supplying tools for RNA complex prediction.

**Figure 7:**
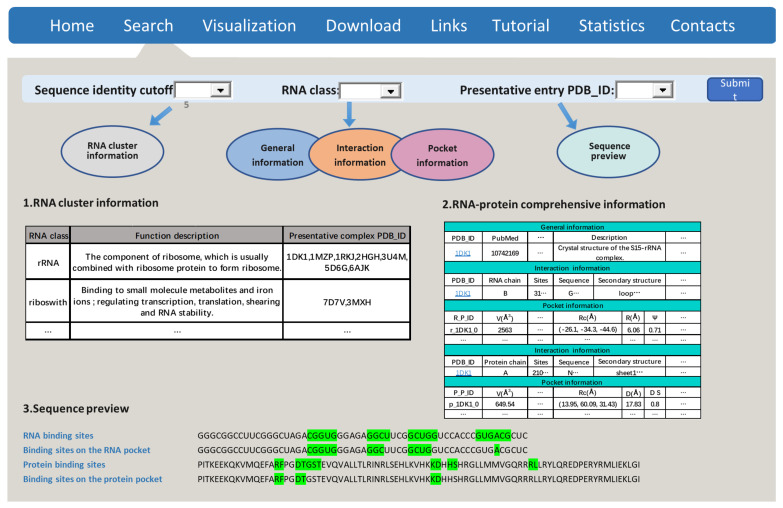
The search module of RPpocket. The user interface provides the RNA cluster, RNA–protein pocket topology, and sequence motif patterns. The sequence colored in green shadow represents the RNA-protein interaction or binding sites on the pocket.

RPpocket has eight modules: Home, Search, Visualization, Download, Links, Tutorial, Statistics, and Contacts. The Home module mainly introduces the RPpcoket database and the navigation to other modules (Figure S1). The search module consists of four parts (Figure 7): a drop-down selection box, a table of RNA descriptions (Figure S2), a summary table of RNA–protein complexes, and a sequence preview module (Figure S3). Users can select the pocket by the RNA class drop-down selection box, choose a complex from the entry representative, and click the submit button to obtain the relevant information about the complex. In the visualization module, users can upload and investigate the structure. The structure will be visualized in four representations: “spacefill”, “wire”, “ball&stick”, and “cartoon”. Users can zoom and rotate the structure, generate and save pictures. The download module provides pocket-based interactions for each RNA–protein complex. Users can download the information in xlsx format and the structures in MRC or PDB formats. The link module provides resources for RNA-related simulation, prediction, and databases. The Tutorial module offers the RPpocket introduction and the abbreviation for the RPpocket database. Data analysis results are provided in the Statistics module. The Contacts module provides emails for users to comment or ask questions.

## 3. Discussion

The NDB (Nucleic Acid Database) and PDB (Protein Data Bank) are widely used databases for biologists [12,13]. The NDB provides information about experimentally determined nucleic acids and complex structures. The PDB includes archive information with the tertiary shapes of proteins, nucleic acids, and complex structures. The NDB and PDB provide a structural view of biology. However, both databases do not provide detailed interaction features between RNA and protein.

Currently, several databases focus on RNA–protein complexes (Table S5). NPInter is a database that collects RNA-related structural interactions by integrating published literature and processing high-throughput sequencing. It provides detailed annotations and predicted interaction scores for complexes [17]. RNAInter integrates experimental validation and computational predictions of RNA interaction data from the literature and 35 other resources [27]. In addition, it provides interaction annotations, including RNA modification sites and RNA subcellular localization [15]. Unfortunately, these databases do not perform detailed calculations of the RNA pockets. For example, HDOCK used three published protein–RNA benchmarks as testing sets. The protein–RNA docking benchmark 1.0 by the Zou group [28], the protein–RNA docking benchmark v1.1 from the Fernandez-Recio group, and the protein–RNA docking benchmark version 2 from the Bahadur group [29,30]. The NPDOCK used 12 protein–RNA complexes from the Varani and Fernandez benchmarks [31,32].

Several existing protein-related databases also provide sequence, structure, or interaction information (Table S5). For example, (1) HIPPIE, Tissue Net, and MyProtein Net provide tertiary structure information [33,34,35], and (2) the BioGRID provides interaction motif information [36]. However, well-analyzed pocket and binding sites are still needed, especially when targeting the pockets in RNA for drug development. We plan to update the database every two years.

## 4. Materials and Methods

### 4.1. Construction of RNA–Protein Dataset

To construct the RNA–protein dataset (Figure S4), we used the search options “Entry Polymer Types” and “Protein/NA” in the structure attribute of the advanced search page of the Protein Data Bank before 22 March 2021, then added the additional condition of “Structure Keywords” and “RNA”, which means that our dataset includes complexes containing only RNA and protein. We extracted the RNA–protein structures with a single RNA chain binding to proteins with chains ranging from 1 to 10. Moreover, the RNA–protein complex structures were composed of 20~500 nucleotides or amino acids. The first structural model was selected if the RNA had several NMR structures. Then, we used the CD-hit server to discard all the RNA–protein complexes with RNA sequence identities > 95% [37,38]. Thus, 74 RNA–protein complexes were left to construct the dataset. Among them, 50 structures were obtained by X-ray diffraction, 23 structures by NMR, and 1 by electron microscopy.

### 4.2. Pocket and Binding Site Identification

An RNA–protein interface was defined if the heavy atom distance between nucleotide and amino acid was less than 4 Å. We extracted the secondary structure in dots and brackets format from the RNA FRABASE 2.0 [39]. The secondary structure and tertiary structure visualizations are shown by TBI-forna and PyMOL [40,41].

The RNA pockets were detected using a rolling probe method with the 3V program [42,43,44,45]. This extracts the volume quickly by taking the difference between the surface of the solvent with two rolling probes. We used the default outer (10 Å) and inner (3 Å) probe radius for volume detection. The sphericity ψ represents the similarity between the pocket and the standard sphere of the pocket, ranging from 0 to 1. A higher value indicates a more standard sphere. The following formula defines the sphericity:

ψ=π136V23A

where *V* and *A* represent the volume and area of the pocket [45,46].

The protein pockets were detected by DoGSiteScorer, which is widely used for detecting protein surface pockets and subpockets [47,48,49,50]. The DoGSiteScorer uses heavy atom coordinates to detect the pockets on the protein surface, provide the pocket mesh shape, and mark the grid points. Then, DoGSiteScorer calculates the volume and surface area values by multiplying the number of mesh points.

## 5. Conclusions

We statistically analyzed RNA–protein complex sequences, secondary structures, and topological pocket features in this work. The results show that protein loops prefer to bind to the RNA stem region. Long-range electrostatic interactions bring RNA and protein together. Short-range interactions optimize the complex. We also developed one easy-to-use database, RPpocket, to provide pocket-based topological interaction information. We hope that RPpocket will facilitate RNA-related study and inhibitor design.

## Data Availability

RPpocket is available at http://zhaoserver.com.cn/RPpocket/RPpocket.html, accessed on 23 May 2022.

## Supplementary Materials

The following are available online at https://www.mdpi.com/article/10.3390/ijms23136903/s1.
