# Supplementary material for: RPpocket: An RNA–Protein Intuitive Database with RNA Pocket Topology Resources
Source: Int J Mol Sci. 2022 Jun 21;23(13):6903. doi: 10.3390/ijms23136903 (PMC9266927; doi:10.3390/ijms23136903)
Supplement: Supplementary file 1 [file ijms-23-06903-s001.zip › ijms-1762183-supplementary.pdf]

Supplementary Information

RPpocket: An RNA–Protein Intuitive Database with RNA Pocket Topology Resources

Rui Yang †, Haoquan Liu †, Liu Yang, Ting Zhou, Xinyao Li and Yunjie Zhao *

Institute of Biophysics and Department of Physics, Central China Normal University, Wuhan 430079, China; ryang@mails.ccnu.edu.cn (R.Y.); liuhaoquan@mails.ccnu.edu.cn (H.L.); YL2019212120@mails.ccnu.edu.cn (L.Y.); tzhou@mails.ccnu.edu.cn (T.Z.); lixinyao@mails.ccnu.edu.cn (X.L.)

* Correspondence: yjzhaowh@mail.ccnu.edu.cn

† These authors contributed equally to this work.

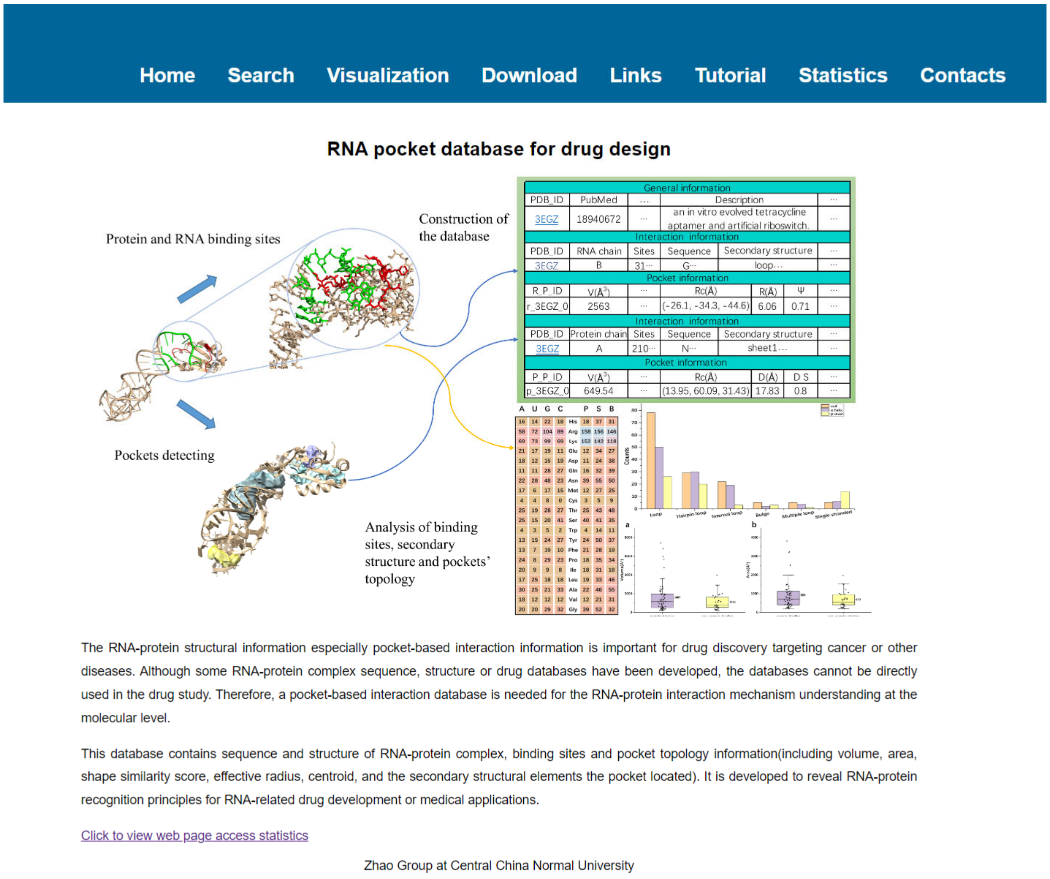

Figure S1. The sketch of the Home module. The Home module introduces the RPpocket database and navigation to other database modules.

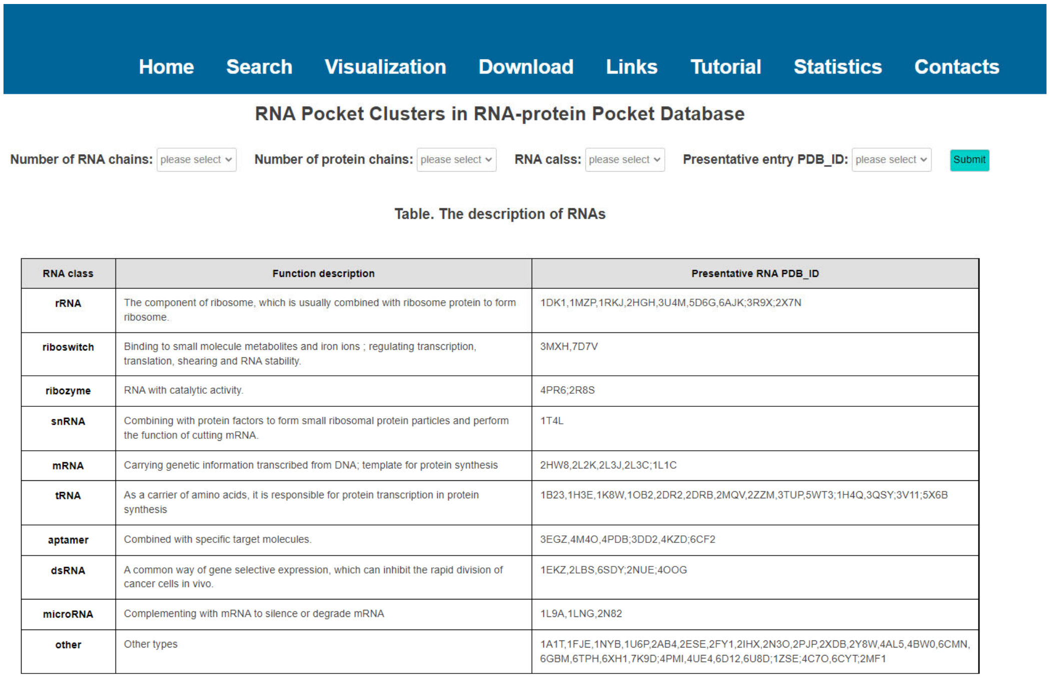
Home Search Visualization Download Links Tutorial Statistics Contacts

RNA Pocket Clusters in RNA-protein Pocket Database

Number of RNA chains: please select Number of protein chains: please select RNA calss: please select Presentative entry PDB_ID: please select Submit

Table. The description of RNAs

RNA class	Function description	Presentative RNA PDB_ID
rRNA	The component of ribosome, which is usually combined with ribosome protein to form ribosome.	1DK1,1MZP,1RKJ,2HGH,3U4M,5D6G,6AJK;3R9X;2X7N
riboswitch	Binding to small molecule metabolites and iron ions ; regulating transcription, translation, shearing and RNA stability.	3MXH,7D7V
ribozyme	RNA with catalytic activity.	4PR6;2R8S
snRNA	Combining with protein factors to form small ribosomal protein particles and perform the function of cutting mRNA.	1T4L
mRNA	Carrying genetic information transcribed from DNA; template for protein synthesis	2HW8,2L2K,2L3J,2L3C;1L1C
tRNA	As a carrier of amino acids, it is responsible for protein transcription in protein synthesis	1B23,1H3E,1K8W,1OB2,2DR2,2DRB,2MQV,2ZZM,3TUP,5WT3;1H4Q,3QSY;3V11;5X6B
aptamer	Combined with specific target molecules.	3EGZ,4M4O,4PDB;3DD2,4KZD;6CF2
dsRNA	A common way of gene selective expression, which can inhibit the rapid division of cancer cells in vivo.	1EKZ,2LBS,6SDY;2NUE;4OOG
microRNA	Complementing with mRNA to silence or degrade mRNA	1L9A,1LNG,2N82
other	Other types	1A1T,1FJE,1NYB,1U6P,2AB4,2ESE,2FY1,2IHX,2N3O,2PJP,2XDB,2Y8W,4AL5,4BW0,6CMN,6GBM,6TPH,6XH1,7K9D;4PMI,4UE4,6D12,6U8D;1ZSE;4C7O,6CYT;2MF1

Figure S2. An example of the Search module. The Search module consists of a pulldown search box and a summary table of RNA clusters. (1) In the pulldown search box, users can select the object by sequence identity cutoff, RNA class, and presentative RNA PDB ID. (2) The RNA description table shows the representative RNAs based on their functions.

1. General information

Table1. General information of 1DK1

General information							
PDB_ID	PubMed	RNA class	RNA Nts	Protein length	Method	Re(Å) [1]	Description
1DK1	10742169	rRNA	57	86	X-RAY DIFFRACTION	2.8	Crystal structure of the S15-rRNA complex.

2. Pocket information of 1DK1

Table2. Pocket information of RNA chain

Interaction surface information								
PDB_ID	RNA chain	RNA binding sites		Sequence	Reported RNA secondary structure?	Secondary structure of the binding sites		
1DK1	B	20-24;30-32,33;37-39,40-41;49-50,51-52,53,54		CGGUG;GGC,U;GCU,GG;GU,GA,C,G	Yes	stem1;stem2,hairpin loop;stem2,internal loop,stem3,bulge,stem4,stem5		
Pocket information								
R_P_ID[2]	V(Å^3)[3]	A(Å^2)[4]	R_c (Å)[5]	R(Å)[6]	ψ[7]	Binding sites	Sequence	Secondary structure[8]
r_1DK1_0	304	270	(-41.6, -42.4, -36.0)	3.37	0.81	52	A	bulge
r_1DK1_1	1529	885	(-39.4, -35.6, -32.6)	5.18	0.72	20-24,30-32,37-40	CGGUG,GGC,GCUG	stem,internal loop
r_1DK1_2	472	328	(-42.3, -42.2, -34.5)	4.31	0.89	20	C	stem
r_1DK1_3	192	189	(-44.3, -40.7, -36.7)	3.05	0.85	N/A	N/A	N/A

Table3. Pocket information of protein chain

Interaction surface information												
PDB_ID	protein chain	Protein binding sites			Sequence					Secondary structure of the binding sites		
1DK1	A	116-117;120-123,124;147-148;150-151;164-165			RF;DTGS,T;KD;HS;RL					loop1;loop1,helix1;loop2;helix2;helix2		
Pocket information												
P_P_ID[9]	V(Å^3)	A(Å^2)	R_c (Å)	R(Å)[10]	D(Å)[11]	enclose[12]	c/a[13]	b/a[14]	D S[15]	Binding sites	Sequence	Secondary structure
p_1DK1_0	406.27	746.61	(10.00, 50.17, 7.92)	14.19	13.45	0.18	0.05	0.18	0.65	116-117,120-121	RF,DT	loop
p_1DK1_1	248.19	572.59	(12.65, 49.22, 23.29)	11.12	10.45	0.17	0.19	0.1	0.45	N/A	N/A	N/A
p_1DK1_2	157.12	422.91	(21.03, 45.50, 32.00)	6.47	10.69	0.31	0.15	0.43	0.32	147-148	KD	loop
p_1DK1_3	138.32	229.05	(7.94, 40.22, 5.76)	6.45	8.78	0.26	0.34	0.83	0.28	116	R	loop
p_1DK1_4	119.74	281.98	(12.48, 38.32, 15.53)	5.53	10.74	0.06	0.26	0.32	0.34	N/A	N/A	N/A

Note. [1] Re: resolution of the structure; [2]R_P_ID: RNA pocket ID; [3] V: volume of the pocket; [4] A: surface area of the pocket; [5] R_c: center of mass of the pocket; [6] R: effective radius of the pocket; [7] ψ: a measure of how much the pocket resembles a sphere; [8] Secondary structure: secondary structure of RNA/Protein located on the pocket; [9] P_P_ID: protein pocket ID. [10] R: maximum radius of the pocket;[11] D: depth of the pocket.[12] enclose: ratio of number of surface to hull grid points;[13] c/a: ellipsoid main axes ratios, with a > b > c;[14] b/a: ellipsoid main axes ratios, with a>b>c;[15] D S : DrugScore for protein pocket

Click to view the interaction graph of RNA-protein

3. Sequence Preview

**RNA binding sites**	GGGCGGCCUUCGGGCUAGACGGUG GGAGAGGCUUCGGCUGGUCCACCCGUGACGCUC
**Binding sites on the RNA pocket**	GGGCGGCCUUCGGGCUAGACGGUG GGAGAGGCUUCGGCUGGUCCACCCGUGACGCUC
**Protein binding sites**	PITKEEKQKVMQEFARFPGDTGSTEVQVALLTLRINRLSEHLKVHKKDHHSHRGLLMMVGQRRRLLRYLQREDPERYRMLIEKLGI
**Binding sites on the protein pocket**	PITKEEKQKVMQEFARFPGDTGSTEVQVALLTLRINRLSEHLKVHKKDHHSHRGLLMMVGQRRRLLRYLQREDPERYRMLIEKLGI

Figure S3. An example of RNA 1DK1 search result. The search results include (1) The representative RNA and members in the RNA cluster; (2) the experiment information and pocket calculation results; (3) RNA/Protein binding sites and binding fragments on RNA/protein pockets in different colors. The sequence colored in green shadow represents the RNA-protein interaction or binding sites on the pocket.

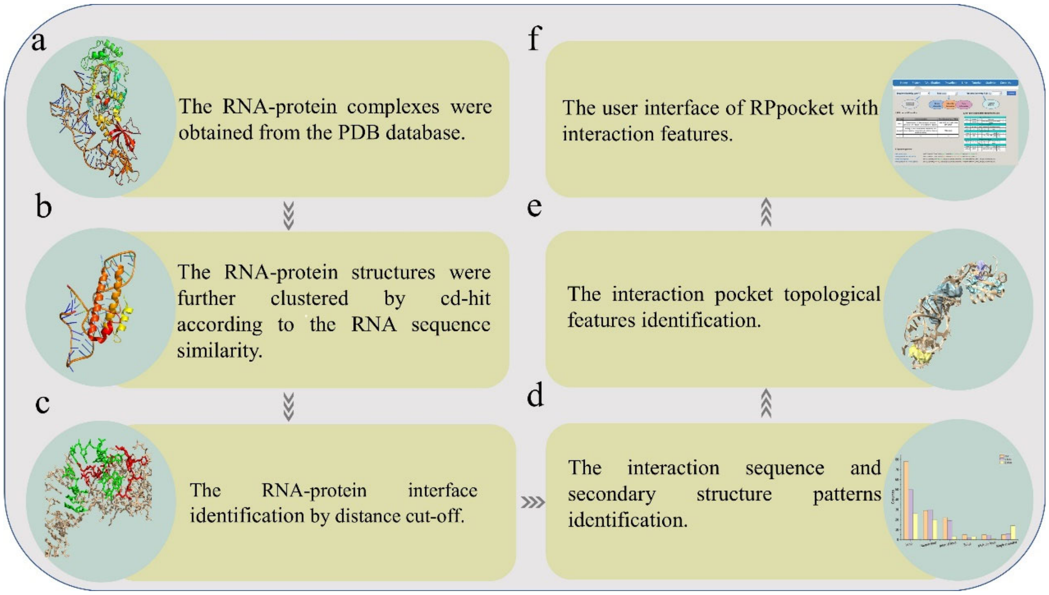

Figure S4. The workflow of the database construction. (a) and (b) The non-redundant RNA-protein complex structures identification. (c)-(e) The detection of sequence, secondary structure, and pocket topological interaction patterns. (f) The user interface of the database.

Table S1. The RNA-protein structural names for different categories.

	RNA class	Number	PDB ID
1	rRNA	9	1DK1,1MZP,1RKJ,2HGH,2X7N,3R9X,3U4M,5D6G,6AJK
2	riboswitch	2	3MXH,7D7V
3	ribozyme	2	2R8S,4PR6
4	snRNA	1	1T4L
5	mRNA	5	1L1C,2HW8,2L2K,2L3J,2L3C
6	tRNA	14	1B23,1H3E,1H4Q,1K8W,1OB2,2DR2,2DRB,2MQV,2ZZM,3QSY,3TUP,3V11,5WT3, 5X6B
7	aptamer	6	3DD2,3EGZ,4KZD,4M4O,4PDB,6CF2
8	ds RNA	5	1EKZ,2LBS,2NUE,4OOG,6SDY
9	micro RNA	3	1L9A,1LNG,2N82
10	other	27	1A1T,1FJE,1NYB,1U6P,1ZSE,2AB4,2ESE,2FY1,2IHX,2MF1,2N3O,2PJP,2XDB,2Y8W,4AL5,4BW0,4C7O,4PMI,4UE4,6CMN,6CYT,6D12,6GBM,6TPH, 6U8D,6XH1,7K9D

Table S2. The sequence and secondary structure information for RNAs.

PDB ID	The sequence and secondary structure information for RNAs
>1A1T	GGACUAGCGGAGGCUAGUCC<br>(((..(((....)))..)))
>1B23	GGCGCGUUAACAAAGCGGUUAUGUAGCGGAUUGCAAAUCCGUCUAGUCC<br>GGUUCGACUCCGGAACGCGCCUCCA<br>(((((((..(((.....[...))).((((.........))))....(((((..]....))))))))))))....
>1DK1	GGGCGGCCUUCGGGCUAGACGGUGGGAGAGGCUUCGGCUGGUCCACCCG<br>UGACGCUC<br>((((.(((....)))..((((((((...(((....)))..))))).)))..)))))
>1EKZ	GGACAGCUGUCCCUUCGGGGACAGCUGUCC<br>((((((((((((((..))))))))))))))
>1FJE	GGCCGAAAUCCCGAAGUAGGCC<br>((((..............))))
>1K8W	GGCAACGGUUCGAUCCCGUUGC<br>.(((((((.......)))))))
>1L9A	GACACUAAGUUCGGCAUCAAUAUGGUGACCUCCCGGGAGCGGGGGACCACCAG<br>GUUGCCUAAGGAGGGGUGAACCGGCCCAGGUCGGAAACGGAGCAGGUCAAAAC<br>UCCCGUGCUGAUCAGUAGUGUC<br>((((((..(.((((((((((..(((((..((((((....))))))..)))))..)))......(((((.....(.((....(((....)))....)).)...))))).))))))).)...)))))
>1LNG	UCGGCGGUGGGGGAGCAUCUCCUGUAGGGGAGAUGUAACCCCCUUUACCUGCCA<br>CCCCGCCAGGCCCGGAAGGGAGCAACGGUAGGCAGGACGUC<br>..((((..(((((.(((((((((....)))))))))..)))))....((((((.....(((.....(((....))).....)))..)))))).))))
>1MZP	GGGAUGCGUAGGAUAGGUGGGAGCCGCAAGGCGCCGGUGAAAUACCACCCUUCC<br>C<br>((((.(......[..(((((..(((....)))..........].)))))).))))
>1NYB	GGUUCACCUCUAACCGGGUGAGCC<br>(((((((((......)))))))))
>1OB2	GCGGAUUUAGCUCAGUUGGGAGAGCGCCAGACUGAAGAUCUGGAGGUCCUGUU<br>GAUCCACAGAAUUCGCACCA<br>(((((((..((((.....[..)))).(((...........))).....(((((..]....))))))))))))....
>1RKJ	GGAUGCCUCCCGAGUGCAUCC<br>((((((((....)).))))))
>1T4L	GGGAUACCAUGUUCAGAAGAACGUGGUAUCUC<br>((((((((((((((....))))))))))))))
>1U6P	GGCGGUACUAGUUGAGAAACUAGCUCUGUAUCUGGCGGACCCGUGGUGGAAUUG<br>AAGUUCGGAACACCCGGCCGCAACCCUGGGAGAGGUCCCAGGGUU<br>.((((..((((((....))))))..)))).....((....(((.(((((((((....)))))....)))))))..))((((((((((....))))))))))
>2AB4	CCACGGUUCGAAUCCGUGGC<br>((((((.......)))))).
>2DR2	GACCUCGUGGCGCAAUGGUAGCGCGUCUGACUCCAGAUCAGAAGGUUGCGUGUU<br>CGAAUCACGUCGGGGUCACCA<br>(((((((.....(.......)....(((((.......))))).....(((((.......))))))))))))....
>2DRB	GGCCCGGGGCGGUUCGAUUCCGCCCUGGGCCACCA

	((((((((((((.......))))))))))))....
>2ESE	GGAGAGGCUCUGGCAGCUUUUCC<br>((((((((((..).)))))))))
>2FY1	GGACUGUCCACAAGACAGUCC<br>((((((((.....))))))))
>2HGH	GGGCCAUACCUCUUGGGCCUGGUUAGUACCUCUUCGGUGGGAAUACCAGGUGCC<br>C<br>((((....((....))(((((((.....((.(....).))....)))))))))))
>2HW8	GGGGUGAAGGAGGCUUCGGCCGCGAAACUUCACCCC<br>(((((((((..(((....)))......)))))))))
>2IHX	CUGCCCUCAUCCGUCUCGCUUAUUCGGGGAGCGGACGAUGACCCUAGUAGAGGG<br>GGCUGCGGCUUAGGAGGGCAG<br>((((((((...((((.(((((.......)))))))))....((((.....))))(((....)))...))))))))
>2L2K	GGUAAGGUGGGUGGAAUCCUUCGGGAUCCCACCUACCCUGCC<br>((((.(((((((((.((((....)))).))))))))).))))
>2L3C	GGUAGUAUAACAAUAUCCGUGUUGUUAUAGUACC<br>((((.((((((((((....)))))))))).))))
>2L3J	GGCAUUAAGGUGGGUGGAAUAGUAUAACAAUAUGCUAAAUGUUGUUAUAGUAU<br>CCCACCUACCCUGAUGCC<br>(((((((.(((((((((.(((.(((((((((((.....))))))))))).))).))))))))).)))))))
>2LBS	GGGAUACCAUGUUCAAGUGAACGUGGUAUCUC<br>((((((((((((((....))))))))))))))
>2MQV	GGGCGAGGGUCUCCUCUGAGUGAUUGACUACCCGUCAGCGGGGGUCUUUCAUUU<br>GGGGGCUCGUGCCC<br>(((((.((((((((.....((((..((((.((((....))))))))..))))..)))))))).)))))
>2PJP	GGCGGUUGCAGGUCUGCACCGCC<br>(((((.(((((..))))))))))
>2XDB	AUUCAGGUGAUUUGCUACCUUUAAGUGCAGCUAGAAAUUC<br>....((((....[[[.))))......]]].......
>2Y8W	UCCCCACGCGUGUGGGGAUG<br>.((((((....))))))...
>2ZZM	GCAGGGGUCGCCAAGCCUGGCCAAAGGCGCUGGGCCUAGGACCCAGUCCCGUAG<br>GGGUUCCAGGGUUCAAAUCCCUGCCCCUGCACCA<br>......(..(((.............)))((((((.......))))))(((....)))......((.......))...)......
>3EGZ	GAGGGAGAGGUGAAGAAUACGACCACCUAGGUACCAUUGCACCCGGUACCUAAA<br>ACAUACCCUC<br>(((((.(.((((...........))))((((((((.........)))))))).....).)))))
>3MZH	GGUCACGCACAGGGCAAACCAUUCGAAAGAGUGGGACGCAAAGCCUCCGGCCUA<br>AACCAUUGCACUCCGGUAGGUAGCGGGGUUACCGAUGG<br>...(.((......((...((((((....))))))..[))...(((.((((((((..((.........))))))).]))))))))..[))...)
>3TUP	GCCGAGGUAGCUCAGUUGGUAGAGCAUGCGACUGAAAUCGCAGUGUCGGCGGU<br>UCGAUUCUGCUCCUCGGCACCA<br>(((((((..((((.....[..)))).(((((.......))))).....(((((..]....))))))))))))..

>3U4M	GGGAUGCGUAGGAUAGGUGGGAGCCUGUGAACCCCCGCCUCCGGGUGGGGGGA<br>GGCGCCGGUGAAAUACCACCCUUCCC<br>((((.(......[..(((((..(((......((((((((....))))))))...)))..........].)))))).))))
>4AL5	ACUGCCGUAUAGGCAG<br>.(((((.....)))))
>4BW0	GGGGGAGCCGAAAGGCGAAGAACCCA<br>(((...(((....)))......))).
>4PDB	GGGAUGCUCAGUGAUCCUUCGGGAUAUCAGGGCAUCCC<br>(((((((((...(((((....)))..)).)))))))))

Table S3. Statistical secondary structure analysis for RNA-protein interactions.

RNA class	RNA secondary structure						Protein secondary structure		
	stem	Hairpin loop	Internal loop	bulge	multiple loops	single-stranded	loop	helix	β-sheet
rRNA	60.00%	8.00%	20.00%	8.00%	4.00%	0	36.59%	39.02%	24.39%
riboswitch	0	100%	0	0	0	0	62.50%	0	37.50%
ribozyme	40.00%	40.00%	0	0	0	20.00%	50.00%	16.67%	33.33%
snRNA	50.00%	50.00%	0	0	0	0	50.00%	50.00%	0
mRNA	35.71%	21.43%	42.86%	0	0	0	55.00%	40.00%	5.00%
tRNA	48.15%	33.33%	3.71%	0	3.71%	11.1%	49.40%	27.71%	22.89%
aptamer	66.67%	16.67%	0	16.66%	0	0	47.06%	17.65%	35.29%
ds RNA	50.00%	50.00%	0	0	0	0	46.16%	38.46%	15.38%
micro RNA	36.37%	36.36%	27.27%	0	0	0	45.45%	31.82%	22.73%
other	42.86%	31.43%	5.71%	5.71%	2.86%	11.43%	47.96%	29.59%	22.45%

Table S4. The pocket geometrical information for different RNA categories.

RNA class	Volume ($Å^3$)		Surface area ($Å^2$)	
	non-binding pockets	binding pockets	non-binding pockets	binding pockets
rRNA	224.50±45.96	1309.82±1592.59	224.50±50.20	727.64±679.69
riboswitch	626.25±340.81	2567.00±3090.06	428.00±197.05	1458.50±1511.09
tRNA	1193.80±636.15	1525.93±880.73	739.30±330.26	900.79±431.69
aptamer	2263.50±2430.33	1985.50±816.71	1187.00±1097.43	1021.00±350.72
micro RNA	1711.00±1609.38	3062.60±3055.44	949.00±759.43	1645.40±1445.49
other	1035.89±480.50	1037.80±619.10	630.44±222.94	655.40±308.91
all	1122.91±889.85	1607.03±1605.85	678.94±425.09	923.77±772.47

Table S5. The differences distinguish RPpocket from the existing databases.

Name	Type	Information					Reference
		Sequence	Structure	Binding sites	Motif	Geometrical properties	
HIPPIE	protein-protein	√	√				[1]
Tissue Net		√	√				[2]
MyProtein Net	protein-drug	√	√				[3]
BioGRID		√	√	√	√		[4]
NPInter	RNA-protein	√	√	√			[5]
DBBP		√	√		√		[6]
RNAInter		√	√	√			[7]
RPpocket		√	√	√	√	√	

References

1. Alanis-Lobato G, Andrade-Navarro MA, Schaefer MH; HIPPIE v2.0: enhancing meaningfulness and reliability of protein-protein interaction networks. Nucleic Acids Res. 2017; 45: D408–D414.
2. Barshir R, Basha O, Eluk A, Smoly IY, Lan A, Yeger-Lotem E; The TissueNet database of human tissue protein-protein interactions. Nucleic Acids Res. 2013; 41: D841–D844.
3. Basha O, Flom D, Barshir R, Smoly I, Tirman S, Yeger-Lotem; MyProteinNet: build up-to-date protein interaction networks for organisms, tissues and user-defined contexts. Nucleic Acids Res. 2015; 43: W258–W263.
4. Oughtred, Rose et al. “The BioGRID interaction database: 2019 update.” Nucleic acids Res.2019; 47: D529-D541.
5. Teng X, Chen X, Xue H, Tang Y, Zhang P, Kang Q, et al. NPInter v4.0: an integrated database of ncRNA interactions. Nucleic Acids Res. 2019;48: D160-D165.
6. Park B, Kim H, Han K. DBBP: database of binding pairs in protein-nucleic acid interactions. BMC Bioinformatics. 2014;15 Suppl 15: S5-S13.
7. Lin Y, Liu T, Cui T, Wang Z, Zhang Y, Tan P, et al. RNAInter in 2020: RNA interactome repository with increased coverage and annotation. Nucleic Acids Res. 2019;48: D189-D197.
